# Exposure to Household Air Pollution from Wood Combustion and Association with Respiratory Symptoms and Lung Function in Nonsmoking Women: Results from the RESPIRE Trial, Guatemala

**DOI:** 10.1289/ehp.1408200

**Published:** 2014-11-14

**Authors:** Daniel Pope, Esperanza Diaz, Tone Smith-Sivertsen, Rolv T. Lie, Per Bakke, John R. Balmes, Kirk R. Smith, Nigel G. Bruce

**Affiliations:** 1 Division of Public Health and Policy, University of Liverpool, Liverpool, United Kingdom; 2 Department of Global Public Health and Primary Care, and; 3 Department of Clinical Science, University of Bergen, Bergen, Norway; 4 Division of Occupational and Environmental Medicine, University of California, San Francisco, San Francisco, California, USA; 5 School of Public Health, University of California, Berkeley, Berkeley, California, USA

## Abstract

**Background:**

With 40% of the world’s population relying on solid fuel, household air pollution (HAP) represents a major preventable risk factor for COPD (chronic obstructive pulmonary disease). Meta-analyses have confirmed this relationship; however, constituent studies are observational, with virtually none measuring exposure directly.

**Objectives:**

We estimated associations between HAP exposure and respiratory symptoms and lung function in young, nonsmoking women in rural Guatemala, using measured carbon monoxide (CO) concentrations in exhaled breath and personal air to assess exposure.

**Methods:**

The Randomized Exposure Study of Pollution Indoors and Respiratory Effects (RESPIRE) Guatemala study was a trial comparing respiratory outcomes among 504 women using improved chimney stoves versus traditional cookstoves. The present analysis included 456 women with data from postintervention surveys including interviews at 6, 12, and 18 months (respiratory symptoms) and spirometry and CO (ppm) in exhaled breath measurements. Personal CO was measured using passive diffusion tubes at variable times during the study. Associations between CO concentrations and respiratory health were estimated using random intercept regression models.

**Results:**

Respiratory symptoms (cough, phlegm, wheeze, or chest tightness) during the previous 6 months were positively associated with breath CO measured at the same time of symptom reporting and with average personal CO concentrations during the follow-up period. CO in exhaled breath at the same time as spirometry was associated with lower lung function [average reduction in FEV_1_ (forced expiratory volume in 1 sec) for a 10% increase in CO was 3.33 mL (95% CI: –0.86, –5.81)]. Lung function measures were not significantly associated with average postintervention personal CO concentrations.

**Conclusions:**

Our results provide further support for the effects of HAP exposures on airway inflammation. Further longitudinal research modeling continuous exposure to particulate matter against lung function will help us understand more fully the impact of HAP on COPD.

**Citation:**

Pope D, Diaz E, Smith-Sivertsen T, Lie RT, Bakke P, Balmes JR, Smith KR, Bruce NG. 2015. Exposure to household air pollution from wood combustion and association with respiratory symptoms and lung function in nonsmoking women: results from the RESPIRE Trial, Guatemala. Environ Health Perspect 123:285–292; http://dx.doi.org/10.1289/ehp.1408200

## Introduction

Approximately 2.8 billion people use solid fuels (wood, animal dung, agricultural wastes, charcoal, and coal) for cooking and heating, a number little changed since 1980 (Bonjour et al. 2013; Rehfuess et al. 2006). Solid fuel combustion leads to high levels of health-damaging household air pollution (HAP) including carbon monoxide (CO), particulate matter (PM), nitrogen dioxide (NO_2_), and polycyclic aromatic hydrocarbons (Naeher et al. 2007). Studies consistently show high HAP levels in households using solid fuels, with PM_2.5_ (≤ 2.5 μm) being observed to be 10 to > 50 times the WHO annual average Air Quality Guideline level (WHO 2006). Women and young children especially experience high levels of HAP exposure because of traditional gender-based household roles involving more time in proximity to the stove (Torres-Duque et al. 2008).

Globally, HAP from solid fuel use was estimated by the Global Burden of Disease Project 2010 (GBD-2010) to account for 3.5 million [95% (confidence interval (CI): 2.7, 4.4 million] deaths and 4.3% (95% CI: 3.4, 5.3) of disability-adjusted life years in 2010 (Lim et al. 2012). Additionally it has been estimated that 16% of the 3.1 million deaths from outdoor air pollution are attributable to HAP through its impact on ambient air (Lim et al. 2012). Accordingly, HAP is ranked fourth in terms of global burden when compared with 67 risk factors contributing to the Global Burden of Disease calculations (second among women) (Lim et al. 2012). This HAP-related mortality arises from four disease outcomes: chronic obstructive pulmonary disease (COPD), acute lower respiratory infections (ALRI) in children < 5 years of age, and from cardiovascular disease and lung cancer (Smith et al. 2004, 2014). In addition, although there is a paucity of epidemiological investigation, there is evidence of an association between HAP and other health outcomes including cataracts and adverse pregnancy outcomes (Pope et al. 2010; Smith et al. 2014).

Smith et al. (2014) estimated that HAP-related COPD resulted in almost 800,000 premature deaths per year (Smith et al. 2014). Although cigarette smoking among women remains low in most developing countries, women exposed to HAP in such countries develop COPD with clinical characteristics, quality of life, and increased mortality similar in degree to that of tobacco smokers (Fullerton et al. 2008). Three published systematic reviews and meta-analyses of HAP and COPD have reported significant pooled effect estimates: odds ratio (OR) = 2.80 (95% CI: 1.85, 4.00) (Kurmi et al. 2010), OR = 2.44 (95% CI: 1.90, 2.33) (Hu et al. 2010), and OR = 2.40 (95% CI: 1.47, 3.93) (Po et al. 2011). Recently, a further meta-analysis reported an increased pooled effect of biomass smoke exposure of OR = 1.94 (95% CI: 1.62, 2.33)—an estimate used in the comparative risk assessment of HAP for GBD-2010 (Smith et al. 2014). With most studies included in the meta-analyses not directly measuring exposure, the effect estimates were based on exposure determined by fuel type (e.g., “use of solid fuels” or “exposure to biomass” compared with “use of other fuels”). All meta-analyses identified a larger effect in women, the latter reporting a pooled OR of 2.30 (95% CI: 1.73, 2.06) in women compared with 1.90 (95% CI: 1.15, 3.13) in men, reflecting their greater exposure to HAP (Smith et al. 2014).

These systematic reviews highlighted the variability in study quality and considerable methodological and statistical heterogeneity (Hu et al. 2010; Kurmi et al. 2010; Po et al. 2011). In addition, most studies used cross-sectional or case–control designs (Hu et al. 2010; Po et al. 2011; Smith et al. 2014), with only one retrospective cohort study (Kurmi et al. 2010) and no prospective cohort studies or intervention designs. Finally, only one study used a direct exposure measure (Liu et al. 2007), the rest using exposure proxies (fuel/stove type and time spent by fire) (Hu et al. 2010; Kurmi et al. 2010; Po et al. 2011; Smith et al. 2014). Such proxies can lead to substantial exposure misclassification. Accurate, direct measurement of HAP exposure is required to define exposure–response relationships (Smith-Sivertsen et al. 2009).

A randomized controlled trial (RCT) of a chimney stove intervention in Mexico, ineligible for these systematic reviews due to outcome definition criteria, found a significant reduction in respiratory symptoms and lung function decline when compared with use of open fire only among women (50%) adherent in using the intervention (Romieu et al. 2009). These women had an FEV_1_ (forced expiratory volume in 1 sec) rate decline of 31 mL/year compared with 61 mL/year in open fire users (*p* = 0.012).

The first RCT to investigate the impact of reduced HAP from a chimney cookstove intervention (plancha) on ALRI in children and the respiratory health of their mothers was carried out in rural Guatemala (RESPIRE; Randomized Exposure Study of Pollution Indoors and Respiratory Effects) (Smith et al. 2011). Intention-to-treat analysis found reductions in the risk of respiratory symptoms. In addition, a relationship between symptoms and lung function was found at baseline (Díaz et al. 2007a; Smith-Sivertsen et al. 2009). No significant association with lung function [FEV_1_ and FEV_1_/FVC (forced vital capacity) ratio], however, was observed. Exposure levels, assessed using CO as a proxy for PM_2.5_, were significantly reduced in intervention compared to control (open fire) groups: 61.6%; *p* < 0.0001, although postintervention PM_2.5_ exposure remained high with exposure distributions between groups overlapping (Smith et al. 2011; Smith-Sivertsen et al. 2009). The RESPIRE study allows investigation of the relationship between respiratory outcomes (symptoms and lung function) and a continuous measure of postintervention exposure rather than by intervention and control groups based on the intention-to-treat analysis. Such an analysis would provide further support for the effects of HAP exposure on airway inflammation in this young population.

We present here analysis from the RESPIRE trial modeling HAP exposure as a continuous measure of CO with lung symptoms and function in young nonsmoking women exposed to high HAP levels since birth.

## Methods

The methods for the adult component of the RESPIRE trial have been published (Smith-Sivertsen et al. 2009) and are briefly described below.

*Study population*. Women were recruited from 23 indigenous communities in the rural highlands of San Marcos in northwest Guatemala. At the time of the study the population primarily spoke Mam and illiteracy was common (Hallman et al. 2006).

The main household fuel is wood, typically burned indoors in a three-stone open fire. Average levels of particulate matter (PM_10_- and PM_2.5_-respirable particles; diameter ≤ 10 or ≤ 2.5 μm) in this setting have been measured to be 717 μg/m^3^ and 528 μg/m^3^, respectively (Naeher et al. 2000). Women spend 5 hr/day on average in a room with a lit open fire (Engle et al. 1998). An additional, concentrated source of exposure is the temazcal (traditional sauna) typically taken one to several times per week and leading to very high levels of CO and PM (Thompson et al. 2004). Smoking is uncommon.

A rapid census survey of 5,365 households in this region found 770 houses with children < 4 months and/or a pregnant woman and using open fires eligible for RESPIRE. Of these, 534 were recruited to the study and, following baseline assessment, randomly assigned to an intervention (received the chimney stove) (Smith et al. 2011; Smith-Sivertsen et al. 2009) or control group (open fire). A total of 504 women (mean age, 27.7 years) agreed to participate and were recruited in two phases—phase 1 in October–November 2002 (group A: 153 intervention and 147 control women) and phase 2 in April–May 2003 (group B: 106 intervention and 98 control women).

**Figure 1:**
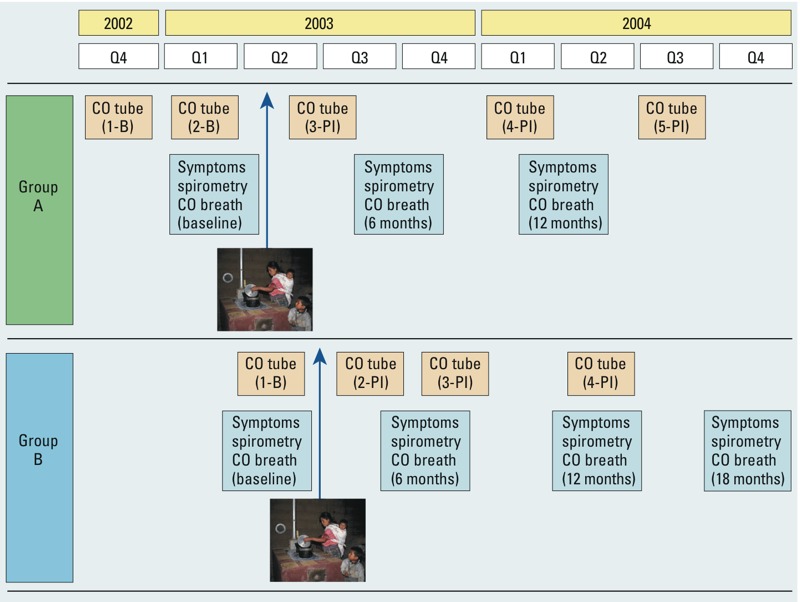
Timing of assessment of personal exposure using CO tubes and of symptoms, lung function, and CO breath, in relation to the installation of the chimney stove, for recruitment groups A and B. Abbreviations: B, baseline; PI, postintervention; Q1–Q4, yearly quarters (3-month intervals). Number of CO tubes is listed (1–5).

The study was carried out over 2 years (October 2002 through December 2004). Women were surveyed at baseline, before randomization, and every 6 months until 12 months (group A) and 18 months (group B) after randomization to intervention and control groups (Figure 1). Questionnaires were administered at each survey by a trained bilingual interviewer. Information included age, height, weight, pregnancy status, smoking, respiratory symptoms, and days since the temazcal was last used. Household information included building construction/layout, number of children, others smoking, and number of consumer goods possessed (radio, television, refrigerator, bicycle, motorcycle, and car) combined into an asset index. Lung function and breath CO were measured at the woman’s home after the interview, and personal 48-hr CO exposure (diffusion tubes) was assessed at different times (Figure 1).

Meteorological data collected over 6 months at the study center in San Lorenzo included rainfall (millimeters per day) and average, minimum, and maximum temperatures (degrees Celsius). These readings were taken for each survey round after completion of interviews. Household elevation was available from baseline GPS measurement.

Ethical approvals for studies involving human participants were obtained from University of California (Berkeley, CA, USA), Universidad del Valle (Guatemala City, Guatemala), the U.S. Centers for Disease Control and Prevention (Atlanta, GA, USA), the University of Liverpool (Liverpool, UK), and WHO (Geneva, Switzerland). Local, trained fieldworkers visited each recruited household and obtained oral informed consent from the study women.

*Assessment of respiratory symptoms and lung function*. Questionnaires recorded frequency and duration of respiratory symptoms including cough, phlegm, wheeze, and chest tightness experienced in the previous 6 months. Cough and phlegm are the most recognized symptoms of airway inflammation, and their chronicity was assessed based on the duration of symptoms (> 3 months indicating chronic cough and/or phlegm). Questions were based on standardized instruments [Medical Research Council/International Union Against Tuberculosis and Lung Disease and International Study of Asthma and Allergies in Childhood (ISAAC)] (Abramson et al. 1991; Bai et al. 1998; ISAAC 1998; Torén et al. 1993), with modification and piloting to improve comprehension (Díaz et al. 2007b). Questionnaires were translated into Mam following Spanish translation, with independent back-translation. A more detailed description of content and development of the study questionnaire has been published (Díaz et al. 2007a, 2007b). For analysis, respiratory outcomes included *a*) presence of cough, phlegm, cough and/or phlegm, wheeze, and chest tightness; *b*) presence of any respiratory symptom; and *c*) presence of chronic cough (> 3 months duration), chronic phlegm, chronic cough, and/or chronic phlegm.

Lung function, without bronchodilator use (FEV_1_, FVC, and FEV_1_/FVC ratio), was measured with a Micro Medical Microloop turbine spirometer (Micro Medical Ltd., Rochester, UK), using American Thoracic Society (1995) guidelines. Quality assurance was maintained through weekly calibration, extensive training, and supervision (Díaz et al. 2007a). Regular assessments of interobserver repeatability were carried out with additional training if required (Díaz et al. 2007a).

*Exposure assessment*. The primary measure of personal HAP exposure was CO (Naeher et al. 2000). Two approaches to assessment were available for this analysis: CO ppm in exhaled breath and 48-hr mean CO ppm measured with passive diffusion tubes.

Measurements of CO breath were carried out immediately following spirometry using a MicroMedical Micro CO (Micro Medical Ltd.). Women performed three measurements outside the home at each survey and the average of the two highest readings was recorded. Quality assurance was maintained through regular instrument comparison, fieldworker training, and supervision (Díaz et al. 2007a). Exhaled CO monitors were calibrated against standard CO 49.8-ppm span gas every 2–4 weeks during the study period (Díaz et al. 2007a; Smith et al. 2010). Previous work carried out by Naeher et al. (2001) in the study region found that CO measurements using the passive diffusion tubes were a good proxy for PM_2.5_ in homes using open fires and plancha stoves with a strong correlation between the measures (*r* = 0.92 for kitchen measurements). In addition when the open fire and plancha data were pooled, a strong correlation between mother personal CO and child personal CO was observed (*r* = 0.85).

Average 48-hr CO ppm was measured using 1DL CO passive diffusion tubes (Gastec Corp., Japan). Quality assurance in tube reading and validation of the data has been reported (Smith et al. 2010, 2011). Tube measurement validation included *a*) an instrument precision substudy (comparing 50 pairs of Gastec 1DL duplicate tube measures), *b*) an exchangeability substudy (comparing 50 pairs of 1D and 1DL tube measures), and *c*) an external validation substudy [where both the 1D (*n* = 45) and 1DL (*n* = 232) tube types were collocated with a continuous electrochemical CO monitor in the household kitchens] (Smith et al. 2010). Exposure measurements using tubes were taken at baseline (before randomization) and three times postintervention until the study end, but did not coincide with the home visits for assessment of symptoms, lung function, and CO in breath (Figure 1). Additional measurements were taken before baseline for group A (Figure 1). Only postintervention measurements were used in the current analysis.

Women were asked to wear the CO tubes during the monitoring period; fieldworkers stressed the importance of keeping the tube on or near the woman at all times, requesting the tube be kept near their bed during sleeping (Smith et al. 2010). The women were asked to remove the tubes when using the traditional sauna (temazcal) because very high levels of CO from this exposure saturate the tube reagent. For CO breath, the relatively short half-life of around 5 hr (Crowley et al. 1989) and the varying timing of measurements relative to recent exposure events of varying intensity at successive surveys will have added further within-person random error. In a study of individual and group variability in exposure assessment for children in the RESPIRE trial, child 48-hr CO was found to have a low reliability for measuring predicted long-term CO exposure through mixed modeling (intraclass correlation coefficient *r* = 0.33) (McCracken et al. 2009).

**Figure 2:**
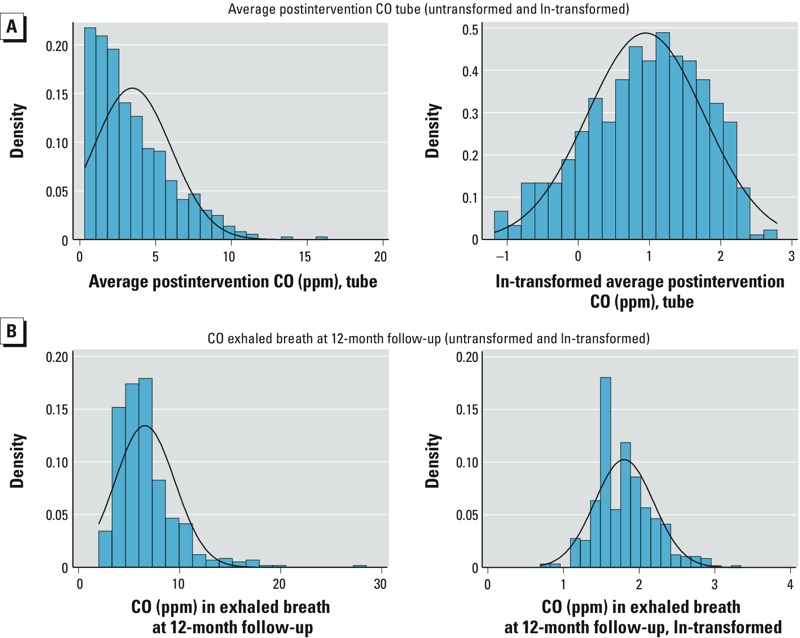
Average postintervention CO tube (untransformed and ln transformed) (*A*). CO exhaled breath at 12-month follow-up (untransformed and ln transformed) (*B*).

*Statistical analysis*. Analysis of exposure in exhaled CO used individual postintervention round measurements because these took place at the same time as the survey interviews and spirometry. Analysis of exposure by CO tubes used the mean of all postintervention measurements for each woman because measurements were not synchronized with home visits for health outcomes (Figure 1). Natural log (ln) transformation of data from both CO measures was used due to marked positive skew (Figure 2A,B).

The relationship between CO measures was assessed by correlation, using Spearman’s rho for untransformed data and Pearson correlation for log-transformed values. For this analysis, we estimated correlations between each CO tube measurement and the breath CO measurement at the next follow-up visit.

We examined the relationship between survey round measurements [ln(CO) exhaled breath] and average postintervention exposure [ln(CO) tube] with lung symptoms experienced in the 6 months preceding each survey using random intercept logistic regression; (xt logit in Stata; StataCorp, College Station, TX, USA). The presence or absence of symptoms during the 6 months before the interview was recorded for each successive survey for repeated measures analysis over the three follow-up periods. Symptoms were defined as chronic if women reported having them for > 3 months.

Because CO in exhaled breath was measured at the same time as spirometry was taken, we examined the relationship between the actual survey round measurements (lnCO ppm) with lung function (FEV_1_, FVC, and FEV_1_:FVC ratio–percentage) as a cross-sectional analysis. For CO measured by the tubes, we modeled average postintervention exposure (lnCO ppm) with lung function. All analysis was conducted using random intercept linear regression (xt reg in Stata).

Confounding covariates were dealt with in two ways. First, factors that were fixed over time or estimated only once over the study were dealt with as non-time-varying covariates [women’s age, height, weight, asset index (McCracken et al. 2009), altitude, and environmental tobacco smoke exposure]. None of the women smoked. Second, factors that varied over time and were re-assessed at each successive survey were dealt with as time-varying covariates by including the survey-specific values. These included *a*) pregnancy status; *b*) season [dry and cold (November–February), dry and warm (March–April), wet (May–October)]; *c*) rainfall (millimeters/24 hr); *d*) day of the week (to account for market days); *e*) daily minimum, maximum, and average temperatures (degrees Celsius); *f*) fieldworker (based on the code for the fieldworker carrying out the interview/spirometry); *g*) recruitment group (coded as A or B; Figure 1); and *h*) days since last temazcal use (this was included as a covariate for CO tube modeling but not for CO breath which incorporates exposure to all recent combustion sources). The covariates were included in multivariable models for adjustment if they modified effect estimates for associations between the exposure variables (CO breath and tube) and the outcome variables (respiratory symptoms and lung function) by at least 5%. For models of lung function, age and height were automatically included.

## Results

Table 1 shows the number of women providing exposure measurements (CO in exhaled breath and CO measured by tube) at each postintervention survey round by recruitment group. In addition, the total number of repeat measurements available for analysis of each CO indicator is shown. For CO measured in breath, > 90% of women provided at least one measurement for both recruitment groups. For CO measured by tube, 93% of women in group A and 88% of women in group B provided at least one exposure measurement. The average/median number of repeat measures was two for CO tubes (for both group A and group B) and two and three for CO breath for groups A and B, respectively. The correlations between CO breath and CO tube measurements taken before each survey were low (Table 2) with *r* values ranging from 0.17 to 0.36. The correlation between average postintervention CO breath and CO tube was low (Spearman’s rho = 0.33; *p* < 0.001).

**Table 1:** Number (%) of exposure measurements available for women postintervention, for CO tube and CO breath by recruitment group and survey round.

	CO tube		CO breath	
	Group A (*n* = 300)	Group B (*n* = 204)	Group A (*n* = 300)	Group B (*n* = 204)
6-month survey	270 (90.0)	65 (31.9)	271 (90.3)	185 (90.7)
12-month survey	196 (65.3)	152 (74.5)	257 (85.7)	181 (88.7)
18-month survey	—	170 (83.3)	—	176 (86.3)
No. of measurements				
0	22 (7.3)	24 (11.8)	25 (8.3)	14 (6.9)
1	90 (30.0)	22 (10.8)	22 (7.3)	9 (4.4)
2	188 (62.7)	109 (53.4)	253 (84.3)	10 (4.9)
3	—	49 (24.0)	—	171 (83.8)

**Table 2:** Pearson correlation of CO tube and CO breath at each postintervention follow-up round (based on ln-transformed values).

Survey round	Recruitment group A		Recruitment group B	
	Coefficient	*p*-Value	Coefficient	*p*-Value
6 months	0.23	0.0002	0.36	0.005
12 months	0.18	0.015	0.17	0.042
18 months	—	—	0.32	< 0.0005

Table 3 shows odds ratios for associations between ln-transformed breath CO at individual visits and symptoms during the previous 6 months, and ORs for associations between symptoms during each 6-month period and the ln-transformed average of all postintervention personal CO measurements. CO breath was positively associated with phlegm, chronic phlegm (> 3 months), cough and phlegm, chronic cough and chronic phlegm (> 3 months), wheezing, and chest tightness, although only univariate associations with wheeze and chronic phlegm were statistically significant (*p* < 0.05).

**Table 3:** Results of random intercept logistic regression for effect of exposure (CO breath, round measurements; and CO tubes, postintervention average values) on respiratory symptoms: all post-intervention survey rounds.

Symptoms	CO breath (*n* = 465; at least one measurement)			CO tube (*n* = 458; at least one measurement)		
	No.^*a*^	OR^*b*^ (95% CI)	*p*-Value	No.^*a*^	OR^*b*^ (95% CI)	*p*-Value
Symptom experienced in the last 6 months
Cough	114 (141)			106 (131)		
Unadjusted		1.01 (0.46, 2.20)	0.98		1.22 (0.88, 1.70)	0.23
Adjusted^*c*^		0.96 (0.44, 2.10)	0.91		1.15 (0.82, 1.61)	0.41
		Age			Age, temazcal	
Phlegm	68 (83)			63 (78)		
Unadjusted		1.82 (0.72, 4.60)	0.21		1.51 (1.01, 2.27)	0.05
Adjusted^*c*^		1.57 (0.61, 4.06)	0.35		1.47 (0.98, 2.21)	0.06
		Age, fieldworker, group, asset,^*d*^ altitude, weight			Age	
Cough or phlegm	135 (169)			126 (158)		
Unadjusted		0.95 (0.45, 2.01)	0.90		1.24 (0.91, 1.69)	0.17
Adjusted^*c*^		0.88 (0.42, 1.86)	0.75		1.20 (0.88, 1.65)	0.25
		Age			Age, fieldworker, group	
Cough and phlegm	46 (55)			42 (49)		
Unadjusted		2.62 (0.90, 7.63)	0.08		1.67 (1.03, 2.72)	0.04
Adjusted^*c*^		2.32 (0.73, 7.40)	0.15		1.63 (1.00, 2.66)	0.05
		Age, fieldworker, group, asset,^*d*^ rainfall, height, weight, pregnancy			Age	
Wheeze	67 (78)			62 (72)		
Unadjusted		2.65 (1.12, 6.26)	0.03		1.64 (1.12, 2.39	0.01
Adjusted^*c*^		2.28 (0.94, 5.52)	0.07		1.57 (1.07, 2.30)	0.02
		Age, fieldworker, asset,^*d*^ altitude, max temp, weight			Age	
Chest tightness	82 (101)			75 (94)		
Unadjusted		2.20 (0.92, 5.25)	0.08		1.38 (0.95, 2.01)	0.09
Adjusted^*c*^		1.71 (0.69, 4.23)	0.25		1.31 (0.90, 1.91)	0.16
		Age, fieldworker, altitude, weight, temazcal			Age	
Any symptom	184 (250)			170 (232)		
Unadjusted		1.32 (0.67, 2.62)	0.42		1.40 (1.04, 1.88)	0.03
Adjusted^*c*^		1.16 (0.57, 2.34)	0.68		1.35 (1.01. 1.81)	0.04
		Age, fieldworker, ETS			Age	
Symptom experienced in the last 6 months for > 3 months
Cough	57 (78)			53 (73)		
Unadjusted		1.45 (0.61, 3.50)	0.40		1.19 (0.84, 1.68)	0.34
Adjusted^*c*^		1.11 (0.46, 2.67)	0.82		1.21 (0.85, 1.74)	0.29
		Age, height, weight, ETS, season, rainfall, min temp			Age, fieldworker, group	
Phlegm	36 (42)			34 (40)		
Unadjusted		3.28 (1.02, 10.54)	0.05		1.37 (0.82, 2.29)	0.23
Adjusted^*c*^		2.56 (0.83, 7.93)	0.10		1.31 (0.77, 2.22)	0.32
		Age, asset,^*d*^ altitude, height, weight			Age, fieldworker, group, height	
Cough or phlegm	73 (85)			69 (80)		
Unadjusted		1.56 (0.67, 3.62)	0.30		1.19 (0.83, 1.69)	0.34
Adjusted^*c*^		1.29 (0.54, 3.09)	0.57		1.21 (0.83, 1.76)	0.32
		Age, fieldworker, height, ETS			Age, fieldworker, group, height	
Cough and phlegm	23 (26)			21 (24)		
Unadjusted		3.15 (0.87, 11.42)	0.08		1.58 (0.87, 2.89)	0.13
Adjusted^*c*^		2.66 (0.66, 10.79)	0.17		1.59 (0.84, 3.01)	0.15
		Age, asset,^*d*^ height, weight, rainfall, pregnancy			Age, height, weight, pregnancy	
ETS, environmental tobacco smoke. ^***a***^Number of women with exposure data reporting a symptom on at least one occasion (number in parentheses is the total number of times the symptom was reported post intervention). ^***b***^Odds ratio for a 1-unit increase in CO exposure (ln transformed), all adjusted for nonindependency of data for repeated measures within individuals. ^***c***^Covariates included in adjusted model are indicated for each analysis. ^***d***^Asset index is based on number of consumer goods possessed (radio, television, refrigerator, bicycle, motorcycle, and car).						

For CO tube measurements, there was a positive association with most respiratory symptoms, with the presence of any respiratory symptom (adjusted OR = 1.35; 95% CI: 1.01, 1.81), phlegm (unadjusted OR = 1.51; 95% CI: 1.01, 2.27), combination of cough and phlegm (adjusted OR = 1.63; 95% CI: 1.00, 2.66), and wheeze (adjusted OR = 1.57; 95% CI: 1.07, 2.30) achieving statistical significance.

For lung function (Table 4), only exhaled CO showed an association, specifically with FEV_1_, with an adjusted value of –35 mL (95% CI: –9, –61) associated with each unit increase in ln-transformed CO breath (adjusted *p* = 0.008).

**Table 4:** Results of random-effects linear regression for effect of exposure [CO breath, all postintervention survey rounds (maximum *n* = 3 per woman); CO tube, postintervention average] on lung function.

Measure of lung function	CO breath (*n* = 465)^*a*^ (round values)		CO tube (*n* = 458)^*a*^ (postintervention average)	
	Coefficient^*b*^ (95% CI)	*p*-Value	Coefficient^*b*^ (95% CI)	*p*-Value
FEV_1_ (L)
Unadjusted	–0.031 (–0.056, –0.006)	0.02	0.012 (–0.030, 0.053)	0.58
Adjusted^*c*^	–0.035 (–0.061, –0.009)	0.01	0.015 (–0.019, 0.050)	0.39
	Age, fieldworker, group, asset,^*d*^ weight, altitude, season, minimum temperature, day of week		Age, height, group, asset,^*d*^ weight, altitude, season, minimum temperature, day of week, temazcal	
FVC (L)
Unadjusted	–0.024 (–0.055, 0.007)	0.13	0.027 (–0.021, 0.075)	0.27
Adjusted^*c*^	–0.026 (–0.057, 0.006)	0.11	0.028 (–0.010, 0.064)	0.14
	Age, weight, altitude, season, minimum temperature, day of week		Age, height, group, asset,^*d*^ weight, altitude, season, rainfall, minimum temperature, day of week	
FEV1:FVC ratio (%)
Unadjusted	–0.401 (–0.963, 0.162)	0.16	–0.319 (–0.853, 0.215)	0.24
Adjusted^*c*^	–0.356 (–0.932, 0.221)	0.23	–0.272 (–0.823, 0.268)	0.32
	Age, height, fieldworker, weight, altitude, season, minimum temperature, day of week		Age, height, group, asset,^*d*^ weight, altitude, season, rainfall, minimum temperature, day of week, temazcal	
^***a***^Of the 465 women providing at least one CO breath measurement, 458 (98%) provided measurements from spirometry. Of the 458 providing at least one CO tube measurement, 431 (94%) provided measurements from spirometry. ^***b***^Beta coefficient represents the difference in lung function in litres associated with a 1-unit increase in CO (ln transformed), all adjusted for time. ^***c***^Covariates are included in adjusted model indicated for each analysis. ^***d***^Asset index is based on number of consumer goods possessed (radio, television, refrigerator, bicycle, motorcycle, and car).				

## Discussion

To our knowledge, this is the first study to examine relationships between a continuous measure of HAP (albeit a proxy for exposure measured by CO) and prevalence of respiratory symptoms and lung function in young, nonsmoking women in a rural biomass fuel–dependent population. Our results found exposure to CO (parts per million in exhaled breath and passive diffusion tubes) associated with common respiratory symptoms, particularly phlegm (tubes), cough and phlegm (tubes), and wheeze (exhaled breath and tubes). Although based on relatively small numbers, more chronic symptoms (> 3 months) appeared to have a larger positive association with CO. In addition, CO in exhaled breath was significantly associated with lower lung function measured at the same time after adjusting for covariates; the average reduction in FEV_1_ for a 10% increase in CO was 3.33 mL (95% CI: –0.86, –5.81).

The average (± SD) breath CO ppm levels in this study were 6.91 ± 3.33, 6.59 ± 2.97, and 6.90 ± 3.42 at 6, 12, and 18 months, respectively. A study of healthy smokers, passive smokers, and nonsmokers found average levels of CO ppm of 17.13 ± 8.50, 5.20 ± 3.38, and 3.61 ± 2.15, respectively (Deveci et al. 2004). The levels observed in these relatively young nonsmoking women therefore fall between those for active and passive smokers and are greater than the published WHO 24-hr air quality guideline for CO of 6 ppm (WHO 2010). Although cigarette smoking is the leading cause of COPD in the developed world, HAP exposure is likely to be an important preventable cause in lower- and middle-income countries, especially in women (Salvi and Barnes 2010).

The global health impact of HAP-related COPD is large, with GBD-2010 estimating that HAP accounted for 783,000 COPD deaths in 2010 (Smith et al 2014). Although there is strong quantitative evidence for an increased risk of COPD from HAP (Hu et al. 2010; Po et al. 2011; Smith et al. 2014), this is largely based on observational data (typically cross-sectional and case–control studies). Increased exposure to biomass combustion has been associated with reduced lung function in cross-sectional studies of populations from Brazil (da Silva et al. 2012), Malawi (Fullerton et al. 2011), and Nepal (Pandey et al. 1985). Our findings provide evidence that may be used to better quantify the respiratory health effects of exposure to HAP.

The strengths of this study are the ability to compare directly measured exposure to a pollutant designated a good proxy for particulate matter (rather than other weaker proxies such as self-reported fuel use or time spent cooking) with objectively assessed lung function measured using standardized spirometry with repeated assessments of exposures and outcomes over 18 months. In addition, information on a range of covariates (both constant and time varying) was available for multivariable modelling of adjusted associations. (Smith et al. 2011).

There did not appear to be a consistent pattern in the relationship between the two measures of CO with respiratory symptoms and lung function. For CO tubes (averaged over the three postintervention periods), there was no association with lung function (reduced FEV_1_); however, for CO breath (analyzed for each of the three postintervention periods), exposure was significantly associated with a lower FEV_1_.

In our relatively young, nonsmoking female population, there were no COPD cases according to Global Initiative for Chronic Obstructive Lung Disease criteria (FEV_1_:FVC < 70) (Lenfant and Khaltaev 2005). Typically HAP-associated COPD is seen in elderly women born in rural areas with lifelong HAP exposure from solid fuel use (Perez-Padilla et al. 2010). Although we did not observe an association between repeated measures of 48-hr personal exposure to CO (as a proxy for PM) and repeated lung function measurements, we did find significant associations with acute and chronic respiratory symptoms, albeit based on small numbers for the latter. In addition, we observed a significant association between CO in exhaled breath (a proxy for more recent exposure) and lower FEV_1_ measured at the same time. Further follow-up of the study women would be needed to fully understand this relationship.

*CO as an indicator for health-damaging constituents of HAP*. CO was used to represent HAP exposure because it was practical to measure lung function using passive diffusion tubes and in exhaled breath (at the same time spirometry was carried out). A wide range of pollutants will contribute to respiratory symptoms and lung function deficit leading to COPD, particularly respirable particulate matter (Ling and van Eeden 2009). Although CO has been demonstrated cross-sectionally to be a reliable proxy for PM_2.5_ in kitchens where biomass is the primary fuel, little is known about this relationship for personal exposure (McCracken et al. 2013). The RESPIRE study quantified the relationship within this study population. Repeated measures (216 measures) of 24-hr PM_2.5_ and CO ppm (passive diffusion tubes) in 116 women found CO explained 78% of between-subject variance in personal PM_2.5_, suggesting that CO is a reliable surrogate for individual’s exposure to respirable particulates (McCracken et al. 2013).

*Mechanisms*. It is likely that HAP exposure causes an inflammatory response and increased oxidative stress in the respiratory tract, particularly in lower airways (Barregard et al. 2006, 2008; Swiston et al. 2008). Evidence from animal models involving rats suggest biomass smoke exposure from cow dung and wood can lead to chronic bronchitis, bronchiolitis, and peribronchial fibrosis (Hu et al. 2013; Rai et al. 1982). Research summarizing the potential adverse health effects of individual constituents of air pollution from woodsmoke identified exposure to nitrogen dioxide as being associated with toxicological effects including pulmonary edema, bronchoconstriction, and increased respiratory infection rates; and exposure to particulate matter as *a*) being associated with decreased lung function in children and *b*) having mutagenic properties within the lower respiratory tract (Pierson et al. 1989). In a controlled experiment where 20 healthy individuals were exposed to woodsmoke combustion with PM_2.5_ levels ranging from 165 to 303 μg/m^3^ and 205 to 662 μg/m^3^ for low and high exposure, respectively, significant mucosal irritation was detected, assessed using a standardized self-reported rating scale of irritation of the throat, nose, and eyes (Riddervold et al. 2011). Acute exposure to particulate matter from ambient air pollution episodes has been found to be associated with reduced lung function in children in the United States and the Netherlands, with declines in lung function associated with episodic exposure to suspended particles occurring rapidly and persisting for up to 3 weeks before recovery (Pierson et al. 1989). In a U.S. study of exposure to ambient particulate matter and respiratory effects in asthmatic children, levoglucosan (a marker of woodsmoke) was measured outside and was found to be significantly associated with a decrease in measured lung function (FEV_1_) (Allen et al. 2008). In a sample of 45 women from the RESPIRE population, we found exposure to indoor biomass smoke to be associated with higher gene expression of multiple mediators of airway inflammation and remodeling. These mechanisms could explain some of the observed association between prolonged biomass smoke exposure and COPD (Guarnieri et al. 2014). In a study comparing COPD patients exposed to woodsmoke and tobacco smoke with controls for matrix metalloproteinase (MMP) activity and expression, researchers found significant increases in MMP-2 and MMP-9 in both exposed groups and concluded that such increased activity from wood smoke exposure could produce lung damage similar to COPD associated with tobacco smoke (Montaño et al. 2004). In addition, an imbalance of oxidants/antioxidants caused by pollutants such as those derived from biomass combustion could play a role in COPD pathogenesis by regulating redox-sensitive transcription factors (e.g., nuclear factor κB), autophagy, and unfolded protein response leading to chronic lung inflammatory response (Yao and Rahman 2011). CO has been implicated as a possible indicator of lung and/or systemic inflammation, and breath CO has been widely studied as a putative inflammatory marker of disease in relation to asthma, COPD, cystic fibrosis, bronchiectasis, and systemic conditions (sepsis and diabetes) (Ryter and Choi 2013). Our study identified an increased risk of common and chronic respiratory symptoms with higher CO exposure measured by tube and in breath (although the majority only reached statistical significance for the former), consistent with acute mucosal irritation and inflammatory response.

There are a number of limitations in the current study that should be considered when interpreting the findings. First, given the observational nature of the analysis (despite being part of a randomized trial), there is likely to be some confounding. We attempted to account for this by adjusting for a variety of covariates collected as part of the trial; however, there is the potential for residual confounding or confounding where the covariates were not adequately controlled. Second, there might be some differential misclassification between women who received the intervention (less exposed) and those who were controls in terms of the self-reporting of respiratory symptoms. Finally, for some of the respiratory outcomes confidence intervals were wide, reflecting low precision due to small numbers of women reporting symptoms (particularly those that were chronic).

## Conclusion

RESPIRE Guatemala (the first RCT studying the effect of HAP on health) has allowed examination of relationships between directly measured personal HAP exposure (CO ppm) and risk of respiratory symptoms and levels of lung function in young, nonsmoking women—evidence previously missing from the literature. Common respiratory symptoms (including cough, phlegm, wheeze, and chest tightness) were increased in association with CO concentrations measured in exhaled breath and by passive diffusion tube. Chronic symptoms (> 3 months) were also associated, although based on a small number of observations. In addition, CO in exhaled breath was significantly associated with lower lung function at the same time, after adjusting for covariates with an average reduction in FEV_1_ for a 10% increase in CO of 3.33 mL (95% CI: –0.86, –5.81). Given the limitations of our study in relation to the potential for uncontrolled confounding, misclassification, and random error, confirmation of these findings requires further prospective studies to examine changes in COPD incidence following interventions to decrease HAP exposure from solid fuel use, and will require larger sample sizes and/or duration of follow-up.
