# Erratum: “Exposure to Household Air Pollution from Wood Combustion and Association with Respiratory Symptoms and Lung Function in Nonsmoking Women: Results from the RESPIRE Trial, Guatemala”

**DOI:** 10.1289/ehp.124-A48

**Published:** 2016-03-01

**Authors:** Daniel Pope, Esperanza Diaz, Tone Smith-Sivertsen, Rolv T. Lie, Per Bakke, John R. Balmes, Kirk R. Smith, Nigel G. Bruce

Environ Health Perspect 123(4):285–292 (2015), http://dx.doi.org/10.1289/ehp.1408200

The author list of the original paper was incomplete. Anaité Díaz, coordinator of the RESPIRE trial, was listed in the acknowledgments of the paper, but due to her significant contributions to the work, she should have been listed as the sixth author. The correct author lineup and affiliation list for this paper is as follows:

Daniel Pope,^1^ Esperanza Diaz,^2^ Tone Smith-Sivertsen,^2^ Rolv T. Lie,^2^ Per Bakke,^3^ Anaité Díaz,^4^ John R. Balmes,^5,6^ Kirk R. Smith,^6^ and Nigel G. Bruce^1^

^1^Division of Public Health and Policy, University of Liverpool, Liverpool, United Kingdom; ^2^Department of Global Public Health and Primary Care, and ^3^Department of Clinical Science, University of Bergen, Bergen, Norway; ^4^Center for Health Studies, Universidad del Valle de Guatemala, Guatemala City, Guatemala; ^5^Division of Occupational and Environmental Medicine, University of California, San Francisco, San Francisco, California, USA; ^6^School of Public Health, University of California, Berkeley, Berkeley, California, USA

The acknowledgments of the original paper must also be corrected. In addition to moving Anaité Díaz to the author lineup, the work of Eduardo Castro should have been recognized. The correct acknowledgments for this paper should read:

We thank Eduardo Castro for his coordination of the carbon monoxide data collection using the passive diffusion tubes, and John McCracken for his valuable assistance with management and preparation of the carbon monoxide data. We also thank the fieldworkers and staff at the field site for their dedication and cooperation during the study.

Funding for this study was provided by the Norwegian Research Council, with overall funding for the RESPIRE trial from the U.S. National Institute of Environmental Health Sciences (R01ES010178). We acknowledge the support we received from the Guatemalan Ministry of Health.

The authors declare they have no actual or potential competing financial interests.

The authors apologize for these omissions.

